# Crystal and Supramolecular Structure of Bacterial Cellulose Hydrolyzed by Cellobiohydrolase from *Scytalidium Candidum* 3C: A Basis for Development of Biodegradable Wound Dressings

**DOI:** 10.3390/ma13092087

**Published:** 2020-05-01

**Authors:** Lyubov A. Ivanova, Konstantin B. Ustinovich, Tamara V. Khamova, Elena V. Eneyskaya, Yulia E. Gorshkova, Natalia V. Tsvigun, Vladimir S. Burdakov, Nikolay A. Verlov, Evgenii V. Zinovev, Marat S. Asadulaev, Anton S. Shabunin, Andrey M. Fedyk, Alexander Ye. Baranchikov, Gennady P. Kopitsa, Anna A. Kulminskaya

**Affiliations:** 1 Petersburg Nuclear Physics Institute named by B.P. Konstantinov of National Research Center “Kurchatov Insititute”, 1 Orlova Roscha, 188300 Gatchina, Russia; ivanova_la@pnpi.nrcki.ru (L.A.I.); Eneyskaya_ev@pnpi.nrcki.ru (E.V.E.); Burdakov_vs@pnpi.nrcki.ru (V.S.B.); Verlov_na@pnpi.nrcki.ru (N.A.V.); 2 National Research Center Kurchatov Institute, Akademika Kurchatova Sq. 1, 123182 Moscow, Russia; 3 Kurnakov Institute of General and Inorganic Chemistry of the Russian Academy of Sciences, Leninsky pr. 31, 119991 Moscow, Russia; kustinovich@supercritical.ru (K.B.U.); a.baranchikov@yandex.ru (A.Y.B.); 4 Grebenshchikov Institute of Silicate Chemistry of the Russian Academy of Sciences, Adm. Makarova emb. 2, 199155 St. Petersburg, Russia; ltpp@rambler.ru; 5 Frank Laboratory of Neutron Physics, Joint Institute for Nuclear Research, Joliot-Curie str. 6, 141980 Dubna, Russia; Yulia.Gorshkova@jinr.ru; 6 Federal Scientific Research Center “Crystallography and Photonics” of the Russian Academy of Sciences, Leninsky pr. 59, 119333 Moscow, Russia; n_tsvigun@mail.ru; 7 Saint Petersburg Research Institute of Emergency Medicine n.a. I.I. Dzhanelidze, Budapeshtskaya str. 3, 192242 St. Petersburg, Russia; evz@list.ru; 8 Laboratory of Experimental Surgery of Scientific Research Center, Saint-Petersburg State Pediatric Medical University, Litovskaya str. 2, 194100 St. Petersburg, Russia; marat.asadulaev@yandex.ru (M.S.A.); andrej.fedyuk@gmail.com (A.M.F.); 9 Institute of Physics, Nanotechnology and Telecommunications, Peter the Great St. Petersburg Polytechnic University, Polytechnicheskaya str. 29, 195251 St. Petersburg, Russia; anton-shab@yandex.ru

**Keywords:** bacterial cellulose, cellobiohydrolase, enzymatic hydrolysis, meso- and microstructure, wound dressing

## Abstract

The crystal and supramolecular structure of the bacterial cellulose (BC) has been studied at different stages of cellobiohydrolase hydrolysis using various physical and microscopic methods. Enzymatic hydrolysis significantly affected the crystal and supramolecular structure of native BC, in which the 3D polymer network consisted of nanoribbons with a thickness *T* ≈ 8 nm and a width *W* ≈ 50 nm, and with a developed specific surface *S*_BET_ ≈ 260 m^2^·g^−1^. Biodegradation for 24 h led to a ten percent decrease in the mean crystal size *D_hkl_* of BC, to two-fold increase in the sizes of nanoribbons, and in the specific surface area *S*_BET_ up to ≈ 100 m^2^·g^−1^. Atomic force and scanning electron microscopy images showed BC microstructure “loosening“after enzymatic treatment, as well as the formation and accumulation of submicron particles in the cells of the 3D polymer network. Experiments in vitro and in vivo did not reveal cytotoxic effect by the enzyme addition to BC dressings and showed a generally positive influence on the treatment of extensive III-degree burns, significantly accelerating wound healing in rats. Thus, in our opinion, the results obtained can serve as a basis for further development of effective biodegradable dressings for wound healing.

## 1. Introduction

Bacterial cellulose (BC) is known to be synthesized by several Gram-negative strains and one Gram-positive bacterial strain on the air-water interface [1,2]. It is a mechanically strong hydrogel built up with a nanofibril network of cellulose chains forming crystalline (up to 90% vol.) and amorphous (10% vol.) structural fragments (reviewed in [3]). In recent years, a new term “nanocellulose” has appeared, which is increasingly referred to bacterial cellulose, as well [3]. Cellulose produced by bacteria is similar to plant cellulose chemical structure but differs by the absence of such typical plant components as lignin, pectin, and hemicelluloses. The fine-fiber net structure of BC determines its important characteristics: high tensile strength, high flexibility, and elasticity, high water-holding capacity reaching up to 1000% of its dry weight, non-genotoxicity, non-carcinogenicity, and excellent biocompatibility with biological systems [1,3,4]. The high-purity three-dimensional structure of BC nanofibrils stabilized by inter- and intra-fibrillar hydrogen bonds forms a high-strength material. Its Young’s modulus for a single filament equals 118 GPa and is similar to steel and Kevlar^®®^ materials [5]. Such exceptional mechanical characteristics of BC together with the ability to resemble soft tissues under tension [6], are due to decreased diameter of the fiber and increased surface area compared to those in plant cellulose. Over the past 12 years, the unique physicochemical and biological properties of BC have been used to develop hemostatic materials [7], implants [8], artificial blood vessels [9], artificial skin tissue engineering, and skin wound dressings [10,11].

The skin is the largest organ of the human body and performs a huge number of functions from body protection to biosynthesis and storage of various biomolecules (pigments, proteins, etc.) [12]. Therefore, effective therapeutic methods and new biomaterials are demanded for rapid recovery of skin after various damages. Currently, BC is being actively investigated and developed as both an independent and composite material for the treatment of wounds, burns, and ulcers [1,4,13,14]. Despite the obvious advantages of BC in skin tissue engineering and wound healing, its low biodegradability is one of limitations for its wide use as an effective wound dressing [6]. As H. Zhang et al. emphasized in their review [15], one of the key factors for the successful application of natural and synthetic materials in tissue engineering is their biodegradability. Moreover, it is important to control this process because used biomaterials should address variable requirements during the wound healing and tissue or born restoration process. This implies the need to understand the relationship between structure and physical and chemical properties of biomaterials and the necessity in tools that can affect their changes. A controlled technology based on enzymatic hydrolysis of the cellulose being a nature-like way to degrade the polysaccharide, seems to be a good solution to this shortcoming.

In nature, there is a wide range of various enzymes [16,17] that function synergistically to catalyze the disintegration of such a complex polymer as plant (ligno)cellulose-containing biomass consisting primary of lignin and hemicelluloses. In contrast, bacterial cellulose nanofibrils are composed of pure cellulose, therefore only enzymes capable of catalyzing the cleavage of β-1,4-bonds between glucosidic residues in the polymeric chain are required for its hydrolysis. All enzymes involved in this process have a common name “cellulases” and are subdivided onto endo- and exo-acting enzymes [17,18]. Endo-1,4-β-glucanases (or β-(1,4)-D-glucan-4-glucanohydrolases, EC 3.2.1.4) cleave off the cellulose molecule inside the chain in an unordered way forming a set of poly- and oligomeric fragments of various lengths. Exo-enzymes (β-D-glucosidase, EC 3.2.1.21 and cellobiohydrolase (or exo-1,4-β-glucanases, EC 3.2.1.91)) sequentially split off the terminal saccharide or disaccharide residues from poly- or oligosaccharide chains [18]. In the human body, there are no enzymes capable of catalyzing cellulose degradation, though they exist in nature and are well studied at present (reviewed in [18,19]). In 2011, Yang Hu and Jeffrey M. Catchmark reported the introduction of microbial cellulases and beta-glucosidase into the BC in order to develop a biodegradable wound dressing. They studied some practical aspects of the enzyme activity under wound conditions and the integration into BC [20,21]. Since this publication, we failed to find reported progress on the use of enzymatic modifications of BC materials in context with the biodegradation. Obviously, an approach for conducting controlled biodegradation of BC-based material for biomedical applications is necessary. New data on the evolution of the material structure during its hydrolysis could be a good basis for such a technology.

The structure of native bacterial cellulose has been studied in detail by a variety of methods: transmission [22] and scanning [22,23,24] electron microscopy, infra-red- and mass-spectroscopy [25], atomic force microscopy [24,26], X-ray scattering methods [27,28,29], and small-angle neutron scattering techniques [30,31,32]. However, to the best of our knowledge, no data on changes of crystalline and supramolecular structure of a polymer that has underwent enzymatic hydrolysis have been reported, so far. Therefore, the aim of our study was to provide an insight into structural changes that took place during the treatment of the BC material with the well-studied cellobiohydrolase from yeast-like fungus *Scytalidium candidum* 3C (CBHSc) [33]. Here we used a wide range of physical and microscopic techniques including small angle neutron scattering (SANS) and ultra-small angle neutron scattering (USANS), and X-ray diffraction (XRD) analysis as well as atomic force (AFM) and scanning electron microscopy (SEM) to study the CBHSc-catalyzed process of cellulosic nano-gel films degradation in detail. Finally, in order to show the feasibility of the enzyme-BC application in biological systems, we have established the safety of the CBHSc-treated BC in in vitro and in vivo experiments.

## 2. Materials and Methods

### 2.1. BC Sample Production

To prepare samples of BC, the strain *Gluconoacetobacter hansenii* ATCC 10821 was cultured under static conditions in a 250-mL Erlenmeyer flask containing 100 mL of liquid HS medium [34] at 25 °C for 14 days. Then cell suspension was transferred to several flasks with a fresh medium and incubated at a constant temperature of 25 °C for 7 days. To remove bacterial cell debris, BC disks were washed three times with H_2_O, then with a 0.5 M solution of NaOH for 12 h at room temperature with shaking and left in 0.1 M NaOH solution at 60 °C until bleaching. Then the samples were autoclaved and stored in sterile water until use.

### 2.2. Isolation of Cellobiohydrolase from S. Candidum 3C, Activity Assays and Kinetics

Cellobiohydrolase from the fungus *S. candidum* 3C (formerly *Geotrichum candidum* 3C [35]) (CBHSc) that was used for the hydrolysis of BC samples, was purified according to the ref. [33].

Assessment of cellulase activity during protein purification was routinely performed as previously described [33]. CBHSc kinetics experiments were performed in triplicate with BC pieces with an equal size (20 × 20 × 1 mm^3^). To each BC fragment, 300 µL of the enzyme solution (0.5 mg/mL) in 0.1 M sodium acetate (pH 5.0) was added and the samples were kept at 37 °C for 6 h, removing aliquots (from 7 to 50 µL) of the reaction mixture each 15 min. The reaction was stopped by boiling for 5 min and the amount of released reducing sugar in each aliquot was determined with *p*-hydroxybenzoic acid hydrazide reagent as previously described [36], against glucose standards.

### 2.3. BC Samples Preparation for Structural Analysis

For the structural studies, the samples of BC treated with CBHSc were prepared as follows:

- Identical disks of bacterial cellulose (diameter 10 cm, thickness 1 mm) were treated with the enzyme solution (0.5 mg/mL) at 37 °C for 120, 210, 240 min, and 24 h. Then, the hydrolysis was stopped by boiling for 5 min, the samples were washed with distilled water and left in 98% ethanol for 3 days, and alcohol solution was changed once a day.
- The specific setup consisting of a high-pressure pump for CO_2_ (Supercritical 24 (SSI, State College, PA, USA)), a 50-mL steel reactor, and a back-pressure regulator BPR (Waters, Milford, MA, USA) was used for supercritical drying of the obtained BC samples. This drying method was used as the safest procedure to retain the BC supramolecular structure close to its wet-state arrangement [37]. The samples prepared as described above were washed sequentially with supercritical CO_2_ (15 MPa) at 20 °C for 2 h and at 50 °C for 2–2.5 h.

### 2.4. Analytical Methods

X-ray powder diffraction patterns were recorded with an EMPYREAN diffractometer (Malvern Panalytical B.V., Eindhoven, Netherlands) using CuKα incident radiation in the 2θ range 5–50° at a 2θ step of 0.025° and a counting time of 3.5 s per step. The mean particle size (coherent scattering domain size) for cellulose samples was estimated using Scherer equation. Instrumental broadening was established using standard sample LaB_6_.

The morphology of the supercritically dried samples of BC were analyzed by NTEGRA PRIMA microscope (NT-MDT Spectrum Instruments, Zelenograd, Russia). The measurements have been performed in the semi-contact microscopy mode in air at room temperature, fixed needle change of the cantilever NGS01Au (10-nm curvature radius) oscillation amplitude, which determines the surface topography. Images were taken continuously at a scan rate of 0.3 Hz.

The microstructure of supercritically dried BC samples was analyzed on a Carl Zeiss NVision 40 (Carl Zeiss, Oberkochen, Germany) high-resolution scanning electron microscope equipped with an Oxford Instruments X-MAX (Oxford Instruments, Carl Zeiss, Oberkochen, Germany) (80 mm^2^) detector. SEM images were taken with an Everhart-Thornley detector (SE2) at 1 kV accelerating voltage. Before the measurements, the samples were not coated with a conductive layer and were analyzed as is.

The specific surface area *S*_BET_ of the supercritically dried BC samples was measured by the low-temperature nitrogen adsorption method on a QuantaChrome Nova 1200B analyzer (Quantachrome Instruments, Boynton Beach, FL, USA). The samples were degassed at 45 °C in a vacuum for 17 h prior to analysis. Based on the data obtained, the specific surface area *S*_BET_ for the samples was calculated using the Brunauer–Emmett–Teller model (BET) and the seven-points-method within the relative pressure range P/P_0_ = 0.07/0.25 (where P_0_ is the saturation pressure). The calculation of the pore size distribution was carried out on the basis of nitrogen adsorption and desorption isotherms according to the Barrett-Joyner-Halenda method (BJH).

Small angle and ultra-small angle neutron scattering (SANS and USANS) were measured on YuMO facility (IBR-2 pulsed reactor, Dubna, Russia) and KWS-3 facility (FRM_II reactor, Garching, Germany), respectively. The YuMO facility represents a time-of-flight spectrometer operating in a geometry close to point geometry. An incident neutron beam distribution provides an available wavelength range 0.05 ≤ *λ* ≤ 0.8 nm. The use of two ring wire He^3^-detectors [38] at distances of 4 m and 13 m from the sample position allowed measurement of the neutron scattering intensity *I*_S_(*q*) in the momentum transfer range 7 × 10^−2^ < *q* = (4π/*λ*)∙sin(*θ*/2) < 5 nm^−1^, where *λ* is the incident neutron wavelength and *θ* is the scattering angle. Standard data acquisition time per sample was approximately 40 min. The raw data treatment was done using the SAS software package [39]. The measured SANS spectra were corrected considering the scattering from the facility and direct beam, as well as the background, and converted to the absolute scale by normalization to the incoherent scattering cross section of standard vanadium sample. The final SANS curves are presented in the absolute scale with background subtraction [40].

The KWS-3 setup is a high-resolution small-angle diffractometer operating with the use of a toroidal focusing mirror, which allows high resolution of the momentum transfer range up to 1 × 10^−3^ nm^−1^ to be attained [41,42]. The measurements were conducted at a neutron wavelength of *λ* = 1.28 nm (Δ*λ*/*λ* = 0.2). Use of the sample-detector distances SD equal to 1 and 10 m allowed the neutron scattering intensity in the range 2.5 × 10^−3^ < *q* < 1.4 × 10^−1^ nm^−1^ to be measured. Scattered neutrons were recorded with a 2D position-sensitive scintillation 6Li detector (active zone diameter of 8.7 cm with a spatial resolution of 0.36 × 0.39 mm^2^). The raw data were corrected using standard procedures [43] considering the scattering from the direct beam and the facility equipment as well as the background. The resulting 2D isotropic spectra were azimuthally averaged according to the efficiency of the detectors [43] and the sample thickness *L*_s_. The preliminary analysis of USANS data was performed using the QtiKWS software (QtiKWS20-2019-05-06, Garching, Germany) package [44]. All measurements as SANS, and USANS, were carried out at room temperature.

Hence, the use of a combination of these methods (SANS and USANS) enable to obtain a complete pattern of scattering by the supercritical dried samples of BC in the momentum transfer range 2.5 × 10^−3^ < *q* < 5 nm^−1^, which corresponded to analysis of the structure in the range of characteristic dimensions from 1 nm to a few micrometers.

### 2.5. Cytotoxity Evaluation

MTT-tests were performed as described in [45]. A 1 mM stock solution of resazurin sodium salt (Sigma # R7017) diluted in PBS was prepared and sterilized by filtration (0.2 μm pore diameter). Stock solutions were stored at 4 °C for no more than 2 weeks. Working resazurin solutions (100 μM resazurin) were prepared on the same day of use by diluting 1:10 resazurin stock solutions (relative to the total volume) in standard culture media specific for cell type.

For the cell viability test, human glioblastoma primary cell line # 15 from the laboratory cell culture collection [46] was used. Cells were cultured in DMEM/F-12 (1:1) medium, containing L-glutamine and supplemented with 10% fetal bovine serum (BioWest, Nuaillé, France) without antibiotics in 5% CO_2_ at 37 °C. Cells were seeded in 4 parallel 96-well plates, seeding density was set at 1 × 10^4^ cells per well. After 24 h, solid bacterial-derived cellulose (16 mm^3^ per well) and *S. candidum* 3C cellobiohydrolase (0.5 mg/mL) was added.

Statistical analysis was performed using Origin (OriginLab Corporation, Wellesley Hills, MA, USA. Data were analyzed using one- or two-way analysis of variance (ANOVA), followed by post-hoc Tukey multiple comparison. Data are presented as means ± standard error, and *p* < 0.05 was considered significant. Experiments were performed in triplicate and repeated at least three times independently.

### 2.6. Experiments In Vivo

Thirty-two Wystar‒Kyoto male rats (mass 200–250 g) were used in the research. Test subjects were divided into four groups (eight animals each): a group without treatment (control group); a group treated with BC with added CBHSc (BC+CBHSc); a group treated with the commercial Ag-impregnated carboxymethyl cellulose wound dressing purchased in ConvaTec, UK (Aquacel Ag+); a group treated with the same commercial wound dressing with added CBHSc (Aquacel Ag+ + CBHSc). BC dressings in (BC + CBHSc) and (Aquacel Ag+ + CBHSc) groups were treated with 0.25 mg/mL enzyme solution (0.6 mL/pc) and applied to the wound. The present in vivo studies were approved provisionally by the Local Ethics Committee of the Saint-Petersburg State Pediatric Medical University (Protocol #6/8 dated on 27 June 2019). Due to ethical reasons, experimental groups included the minimum amount of test subjects sufficient to obtain reliable results.

A third-degree burn (ICD-10) modeling followed by necrectomy and wound edges fixation with surgical sutures. The burn area was 16 cm^2^, which was about 10% from the overall animal body surface. The wound surface was covered with appropriate dressings. The animals were observed each 7 days for four weeks. All manipulations were performed in condition of diethyl ether general inhalation anesthesia in strict compliance with the provisions of European Convention for the Protection of Vertebrate Animals used for Experimental and other Scientific Purposes (ETS 123). Euthanasia was performed in strict compliance with the Recommendations for Euthanasia of Experimental Animals of European Commission [47,48]. To determine the speed and healing acceleration index, planimetric assessment of the wound surface was used. Healing indices were calculated by the following equation:

(*S* − *S*n) × 100/(*S* × *T*), (1)

where *S* is the previously observed surface area (cm^2^), *S*n is the actual surface area (cm^2^), and *T* is the period between observations (days). The *p*-value was determined by Mann‒Whitney U-test calculations.

## 3. Results

**Figure 1:**
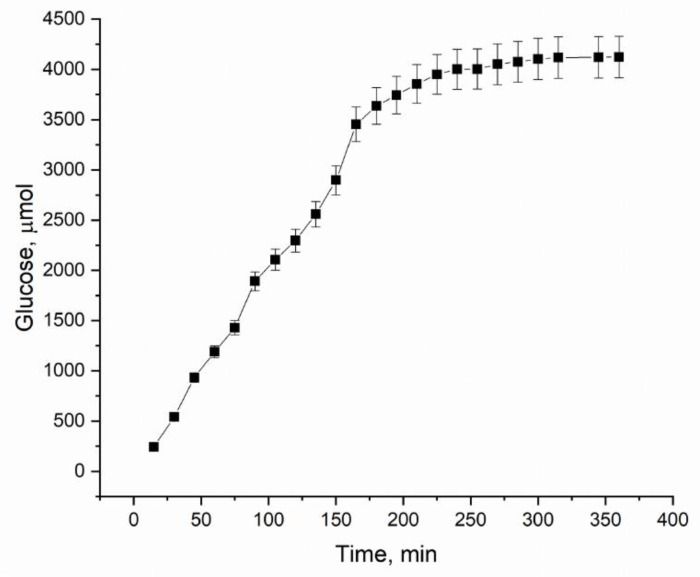
Time-dependent glucose release from bacterial cellulose during the *Scytalidium candidum* 3C (CBHSc)-catalyzed hydrolysis.

The hydrolysis of bacterial cellulose by cellobiohydrolase from *S. candidum* 3C was monitored by time-dependent glucose equivalents release. As shown in Figure 1, the rate of the hydrolysis product release was constant during the first three hours, slowing down sharply between 3 and 5 h of treatment. About 6% of cellulose was degraded after 240 min and 15% after 24 h of the enzymatic treatment. When processing BC samples for 65 h, we achieved 90% conversion of the substrate (data not shown). No glucose equivalents were released in the control sample without the enzyme in the selected conditions.

To study changes of the supramolecular and crystalline structure of the partially hydrolyzed BC, samples were treated with the enzyme for 120, 210, 240 min, and 24 h that corresponds to different parts of the obtained curve: linear growth, the hydrolysis inhibition by product, and a plateau when little glucose equivalent release was observed.

### 3.1. XRD

**Figure 2:**
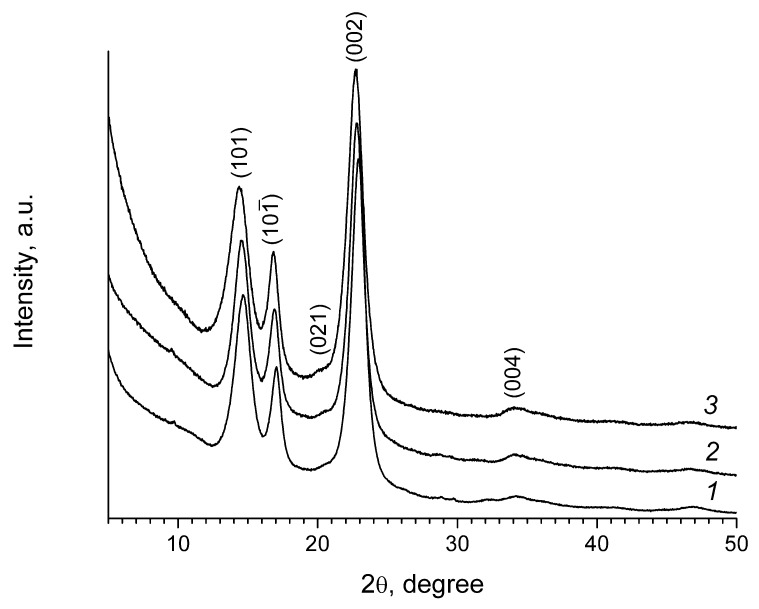
X-ray diffraction patterns of untreated bacterial cellulose (1) and samples treated with CBHSc for 240 min (2) and 24 h (3).

Well resolved reflections at 2*θ* near 14.5°, 16.9°, 22.8°, 34° were seen in the diffraction pattern of cellulose samples without treatment (see Figure 2). According to these data, the structure of the samples can be attributed to highly crystalline cellulose I, most probably Iα allomorph [49]. To index the powder diffraction patterns of bacterial cellulose materials, we followed previously reported data [50,51], though different indexing is possible (see e.g., [52,53,54]).

**Figure 3:**
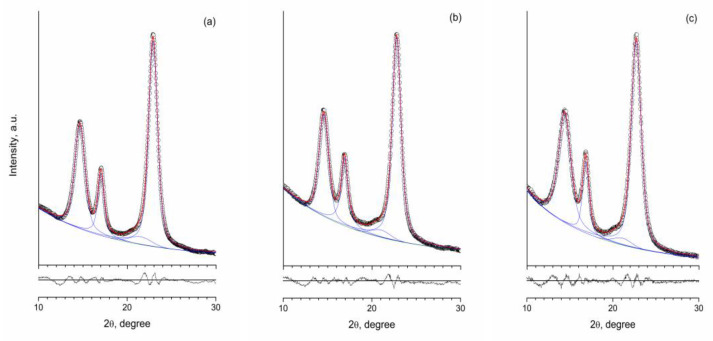
Deconvolution of the diffraction patterns of bacterial cellulose samples to pseudo-Voigt functions. (**a**) an initial sample; (**b**) a sample treated by CBHSc for 4 h; (**c**) a sample treated for 24 h (circles are experimental data, green line is the baseline, red line is the deconvolution, blue line is peak fitting to pseudo-Voigt functions, black line is the difference curve).

The results of the deconvolution of the diffraction patterns of bacterial cellulose samples in the range of 10°–30° 2*θ* to four pseudo-Voigt functions are presented in Figure 3. The estimated mean crystal size *D_hkl_* in the native cellulose sample was 7/8 nm for (101) and (002) reflections and 11 nm for ($10\bar{1}$) reflection and remained virtually unchanged after 4 h of hydrolysis by CBHSc. The sample obtained after 24-h treatment demonstrated an approximately 1 nm decrease in the mean crystal size *D_hkl_* along (101) and (002) planes.

The crystallinity index (CI) was estimated using various techniques [55], but in all the cases the CI was not lower than 90%, indicating the highly crystalline nature of all the materials.

### 3.2. Low Temperature Nitrogen Adsorption

**Figure 4:**
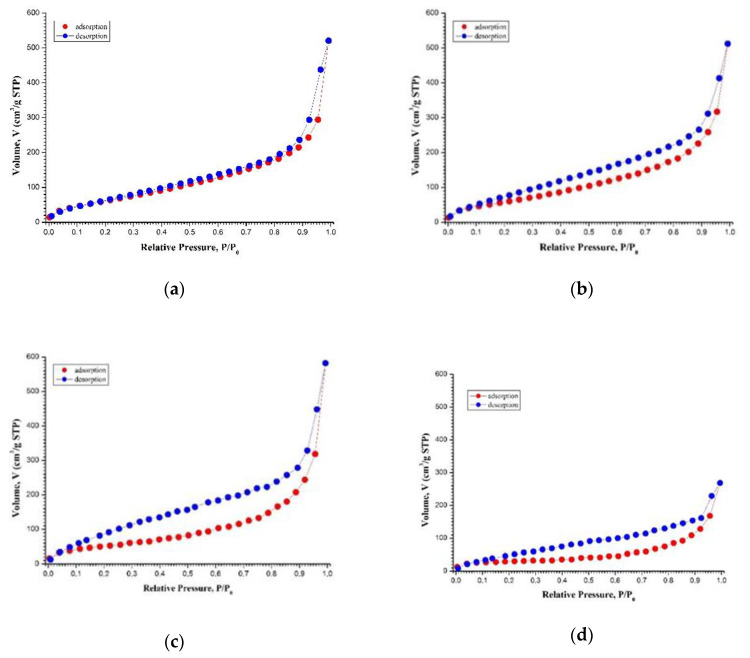
Nitrogen adsorption/desorption isotherms for supercritically dried native nano-gel film (NGF) (**a**) and bacterial cellulose (BC) samples after the biodegradation under the action of cellobiohydrolase from *S. candidum* 3C for 120 (**b**), 240 (**c**) minutes, and 24 h (**d**).

Figure 4 shows nitrogen adsorption/desorption isotherms for supercritically dried samples of the native and CBHSc-treated BC. All presented adsorption/desorption isotherms appear to belong to type IV according to the IUPAC classification [56] and were characterized by type H3 hysteresis, usually associated with the presence of slit pores characteristic to materials consisting of lamellar particles. Type IV isotherms are characteristic of mesoporous materials containing pores where capillary condensation of the adsorbent can occur, which causes the appearance of hysteresis. However, the hysteresis value appeared to depend significantly on the duration of biodestruction. In the case of native BC, the hysteresis was weakly expressed and the hysteresis loop started closing at the relative pressures P/P_0_ > 0.3, which indicated a low content of micropores in this sample.

Full nitrogen adsorption-desorption isotherms for cellulose samples treated by CBHSc differed from the isotherm of the untreated sample. Upon the increase in treatment duration, the hystheresis loop became more pronounced. The adsorption branch of the full isotherm drifted down relative to the desorption branch and its slope decreased in the middle range of nitrogen partial pressures. The latter effect was obviously due to the changes in the porous structure of the material, namely to the decrease in the concentration of mesopores. The former effect was probably due to the formation of the pores with narrow necks.

**Figure 5:**
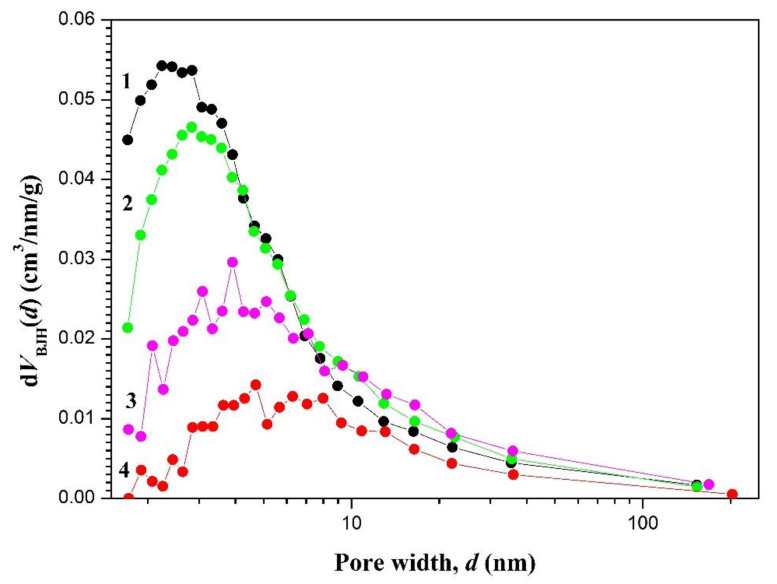
Pore size distributions *d*V(*D*), obtained from the analysis of adsorption isotherms using the Barrett-Joyner-Halenda (BJH) model, for supercritically dried native NGF (1) and BC samples after the biodegradation by cellobiohydrolase from *S. candidum* 3C for 120 (2), 240 (3) minutes, 24 h (4).

The analysis of the changes in pore size distribution indicates (Figure 5) that the treatment by CBHSc resulted in a two-fold increase in the average pore diameter *d* along with almost the same decrease in the mesopore specific volume *V*_P/P0__→0.99_ (from 0.86 to 0.42 cm^3^·g^−1^) and specific surface area *S_BET_* (from ≈260 to ≈100 m^2^·g^−1^).

The results of the analysis of all nitrogen adsorption-desorption isotherms for cellulose samples using BET and BJH models are presented in Table 1 and Figure 4 and Figure 5.

### 3.3. SANS and USANS

Diffraction and adsorption-based methods cannot provide comprehensive information on the structure of materials containing an amorphous phase. Size and shape of scattering inhomogeneities as well as structure of their surface can be obtained using USANS and SANS methods, which are widely used to access the mesostructure of various materials in the 1 nm–1 µm scale range.

**Figure 6:**
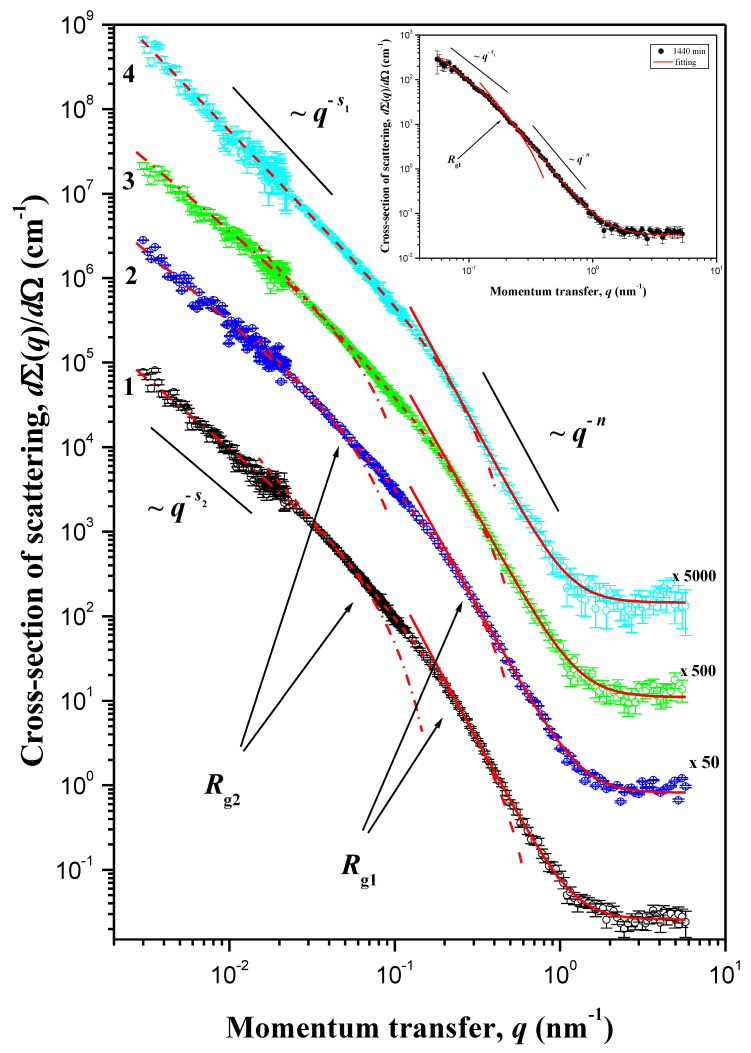
Dependences of neutron scattering cross sections *d*Σ(*q*)/*d*Ω versus the momentum transfer *q* for the supercritically dried samples of BC: native nano-gel film (NGF) (1) and NGF treated by CBHSc for 120 (2), 210 (3), 240 (4) minutes, and 24 h (see insert). For better perception, the *d*Σ(*q*)/*d*Ω values for samples (2), (3), and (4) are multiplied by 5, 15, and 50, respectively. Solid lines are the data fitting in accordance with Equation (3).

Figure 6 shows the experimental log-log plot of neutron scattering cross sections *d*Σ(*q*)/*d*Ω versus the momentum transfer *q* for the supercritically dried samples of BC: native nano-gel film (NGF) and NGF treated by cellobiohydrolase from *S. candidum* 3C for 120, 210, 240 min, and 24 h.

The small angle neutron scattering pattern observed for the supercritically dried samples of BC was typical for the systems with a disordered structure consisting of randomly oriented non-spherical (anisodiametric) objects, for example, for strongly elongated (fibrils) or oblate (lamellas) particles. To describe scattering in the Guinier region, where scattering is determined by the characteristic size *R*_c_ and the shape of independently scattering inhomogeneities, regardless of their local structure, it is necessary to use the generalized expression [57]:

(2) $$\frac{d\Sigma \left(q\right)}{d\Omega }=\frac{G}{q^{s}}\times \exp\left(\frac{-q^{2}R_{g}^{2}}{3-s}\right)$$

where the amplitude *G* is the Guinier pre-factor [58], while the parameter *s* is determined by the shape of the scattering inhomogeneities: *s* equals 0 for spherical objects, *s* equals 1 for one-dimensional particles (fibrils), and *s* equals 2 for two-dimensional inhomogeneities (lamellas) [57]. The values of the parameter *s* can not only be integer, but also fractional.

Since aspherical objects are determined by not only one characteristic size but two sizes (radius *R*_c_ and length *L* in the case of fibrils) or three sizes (thickness *T*, width *W,* and length *L* for lamella), the corresponding Guinier region can contain two or three ranges of momentum transfer *q*, which was entirely in line with the observed experimental data (Figure 6). For supercritically dried samples of BC treated by CBHSc for 0/120 min, three regions could be distinguished on the dependences of the scattering cross sections: the region related to the Porod regime, where scattering was determined by the local structure of scattering inhomogeneities and described by the power dependence; and two regions corresponding to the Guinier regime, where scattering was determined by the characteristic sizes of aspherical scattering inhomogeneities. In the samples of BC treated for 240 min and 24 h (see insert in Figure 6), only one region with the Guinier regime was observed in the corresponding scattering curves. This implies that the radius of gyration *R_g_*_2_, and accordingly, the length *L* in the case of fibrils or width *W* for lamella, exceeds the maximum size of inhomogeneities *R*_max_, the scattering by which can be detected in the experiment at the given resolution of the setup, i.e., in our case *R_g_*_2_ > *R*_max_ ≈ 1150 nm.

The values of the power exponent *n*, determined from the slope of the linear parts of the SANS curves, were within the range from 3.37 to 3.63. The power law exponent in the range 3 < *n* ≤ 4 implies that scattering occurs on the fractal surface with the dimension 2 ≤ *D_S_* = 6 − *n* < 3 [58]. In this regard, the further analysis of scattering in the region *q* < *q_c_* was performed using the two-phase model (solid phase–pore) of the porous structure with the fractal surface of the phase interface [59]. According to this model, an object consists of inhomogeneities (pores) with a strongly developed surface, so that, if the total area of the inhomogeneity (pore) surface measured in the scale of the inhomogeneity (pore) surface size *R* is proportional to *R*^2^, the area of the surface of the scale *r* ≪ *R* is equal to *R*^2^(*R*/*r*) ^Δ^, where 0 < Δ < 1 and *n* = 4 − Δ. In this case, the fractal dimension of the surface, *D_S_* = 2 + Δ, is larger than two.

For *q* > 1.5 nm^−1^, the scattering cross section *d*Σ(*q*)/*d*Ω ceases to depend on *q* and likely corresponds to incoherent scattering on hydrogen atoms in BC making it impossible to examine the scattering process in this region.

In view of this circumstance, to analyze scattering from supercritically dried samples of BC after the biodegradation over the entire *q* range under investigation, we use the generalized empirical Guinier-Porod model [60]:

(3) $$\begin{array}{c} \frac{d\Sigma \left(q\right)}{d\Omega }=\frac{G_{2}}{q^{s_{2}}}\cdot exp\left(\frac{-q^{2}R_{g_{2}}^{2}}{3-s_{2}}\right)\;\mathrm{at}\;q<q_{2},\\ \frac{d\Sigma \left(q\right)}{d\Omega }=\frac{G_{1}}{q^{s_{1}}}\cdot \exp\left(\frac{-q^{2}R_{g1}^{2}}{3-s_{1}}\right)\;\mathrm{at}\;q_{2}<\;q<q_{1},\\ \frac{d\Sigma \left(q\right)}{d\Omega }=\frac{B_{1}}{q^{n1}}+I_{\mathrm{inc}}\;\mathrm{at}\;q>\;q_{1}. \end{array}$$

Here, (3 − *s*_1_) is the dimensional factor; *R_g_*_1_ and *R_g_*_2_ are the characteristic sizes of aspherical scattering inhomogeneities (*R_g_*_1_ < *R_g_*_2_) (for fibrils with the radius *R* and length *L*: *R_g_*_2_ = (*L*^2^/12 + *R*^2^/2)^1/2^, *R_g_*_1_ = *R*/2^1/2^; for lamella with the thickness *T* and width *W*: *R_g_*_2_ = (*W*^2^/12 + *T*^2^/12)^1/2^, *R_g_*_1_ = *T*/12^1/2^); *G*_2_ and *G*_1_ are the Guinier coefficients [58]; *B*_1_ is a coefficient determined by the local structure of scattering inhomogeneities [59]; and *I*_inc_ is a constant determined by incoherent scattering on hydrogen atoms.

To obtain the final results, Equation (3) was convoluted with the instrumental resolution function. The experimental dependences of the differential scattering cross section *d*Σ(*q*)/*d*Ω were processed using the least squares method throughout the entire range under study. The results of the analysis are shown in Figure 6 and Table 2.

As follows from Table 2, all the samples of BC under study were porous systems consisting of anisodiametric inhomogeneities with the fractal surface of the phase interface, and the fractal dimension *D*_S_ of their surface increases with the duration of enzymatic hydrolysis from 2.37 (*t =* 0 min) to 2.63 (*t =* 24 h). The value of parameter *s*_1_ was on the order of two (*s*_1_ = 1.98–2.26), which is indicative of the similarity of their shape to flat objects (ribbons). Estimates of the thickness *T* and width *W* of the nanoribbons in the native nano-gel film of bacterial cellulose were equal to 8.4 and 49 nm, respectively, which is consistent with the estimations of the sizes of nanoribbons obtained in [61,62,63]. The action of cellobiohydrolase led to significant changes in the parameters of the supramolecular structure of the nano-gel film of bacterial cellulose. So, as can be seen from the Table 2, after 24 h of the enzymatic hydrolysis, the thickness *T* of the nanoribbons increased from 8.4 to 13.8 nm, and the width *W* exceeded the maximum size of inhomogeneities, and the scattering that could be detected in the experiment at the given resolution of the setup, i.e., in the case of the treatment for 24 h *W* = (*R*_max_^2^/12 + (*T*/12)^2^)^1/2^ > 330 nm.

### 3.4. AFM and SEM

**Figure 7:**
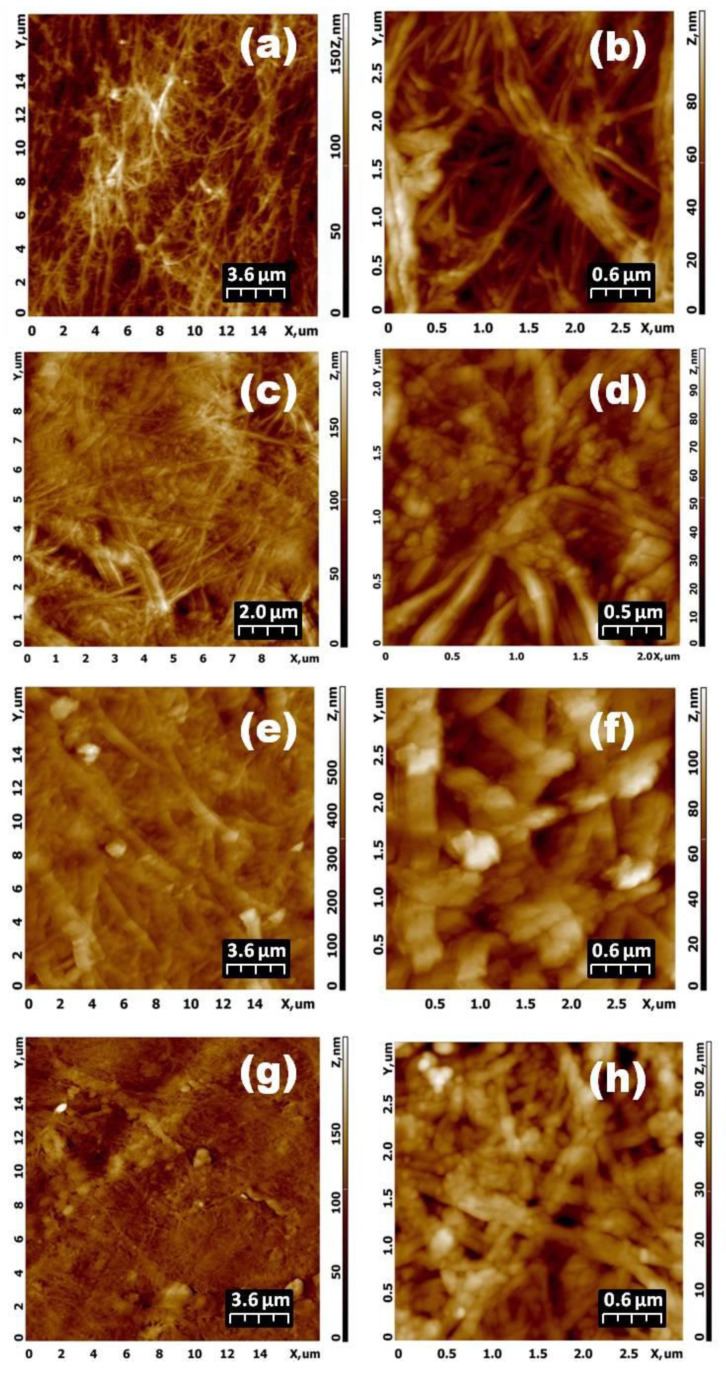
Variation of AFM topologies of the supercritically dried samples of BC: native nano-gel film (**a**): 18 µm × 18 µm, (**b**): 3.2 µm × 3.2 µm, and samples treated by CBHSc for 120 min (**c**): 10 µm × 10 µm, (**d**): 2.5 µm × 2.5 µm, 240 min (**e**): 18 µm × 18 µm, (**f**): 3.2 µm × 3.2 µm and 1440 min (**g**): 18 µm × 18 µm, (**h**): 3.2 µm × 3.2 µm, respectively.

**Figure 8:**
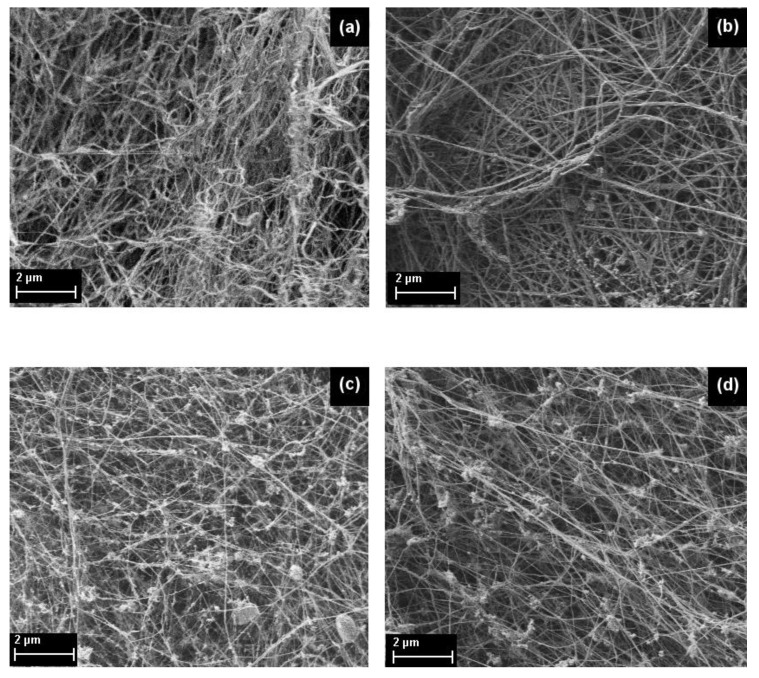
Variation of SEM topologies of the supercritically dried samples of BC: native nano-gel film (**a**) and treated by CBHSc for 120 (**b**), 240 min (**c**), and 24 h (**d**), respectively.

The surface micromorphology of supercritical dried samples of BC treated with cellobiohydrolase from *S. candidum* 3C was studied using AFM and SEM methods (Figure 7 and Figure 8, Table 3).

The morphology of the native nano-gel film of bacterial cellulose (Figure 7a,b and Figure 8a) was a typical 3D polymer network with clearly defined fibers (nanoribbons) with a width of 50/80 nm. Degradation beginning of the 3D polymer network initial structure was clearly seen for the sample treated with CBHSc for 120 min (Figure 7c,d and Figure 8b). Over the entire area of the AFM images, the roughness values had been reducing in comparison with those in the native BC nano-gel film (see the data in Table 3 for 0 and 120 min). However, for individual parts of the sample the Ra and Rq values (indicated in parentheses) differed significantly. The first value was determined at the bottom of the image where the fibril structure had been remained unchanged like in the untreated BC sample. The second one was taken from the top where the structure had significantly been affected by the enzyme, similarly to the sample treated by CBHSc for 24 h (1440 min) (Figure 7g,h). In the sample after 240 min of the reaction, the action of the enzyme extended to the entire surface (Figure 7e,f and Figure 8c). The micromorphology of nano-gel films of bacterial cellulose began changing noticeably. This was expressed, firstly, in a substantial, almost two-fold, broadening of the nanoribbons of the 3D polymer network, and secondly, in the formation of large inhomogeneities (aggregates) in the cells of the polymer network, apparently formed from products (various cellooligosaccharides) from the enzymatic hydrolysis. It is these aggregates that make a significant contribution to increasing the surface roughness. The enzymatic hydrolysis reaction led to an increase in the distance between the nanoribbons of bacterial cellulose, as well as to an increase in the number and size of polymer biodegradation products in 3D mesh cells, as can be clearly seen in the SEM image (Figure 8d). Thus, microscopic studies have confirmed our hypothesis about the “loosening” of the surface of native bacterial cellulose treated with CBHSc.

### 3.5. CBHSc-Toxicity Evaluation of CBHSc-Treatment of BC Samples In Vitro

**Figure 9:**
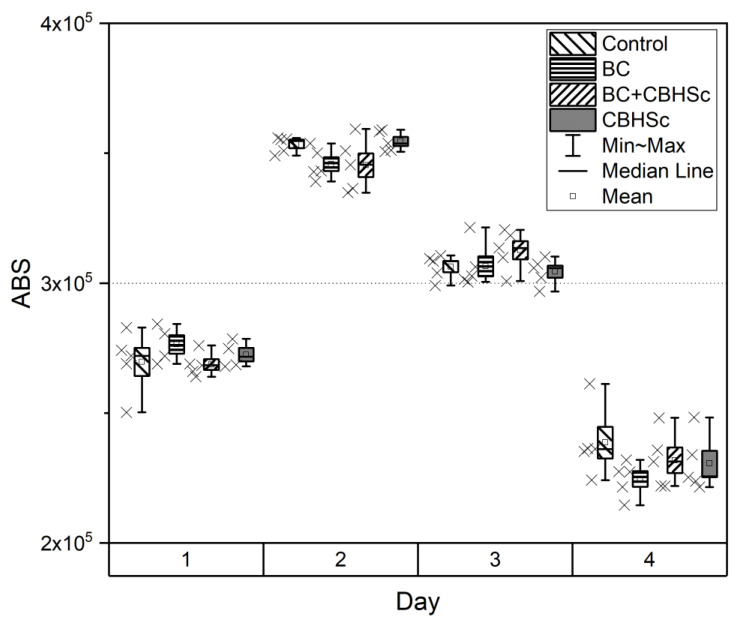
MTT-assay results on the cell proliferation for BC, CBHSc, and BC + CBHSc groups within four days.

To assess the toxic effects of CBHSc and mixture of BC and CBHSc on the cell line of primary human glioblastoma, an MTT-test was used in the experiments. MTT-test (resazurin assay, alamarBlue^®®^ assay) provides a highly effective screening tool that can be used to assess the toxicity, viability, migration, and invasion of mammalian cells, both in early screening of compounds and in the study of the safety and toxicity of drugs and biomaterials [64,65]. We found no significant differences between selected groups (Figure 9) during four days of the observation. Therefore, we concluded that native BC, the solution of CBHSc enzyme, and the BC+CBHSc composition were biocompatible and not toxic in the selected dose range.

### 3.6. Experiments In Vivo

**Figure 10:**
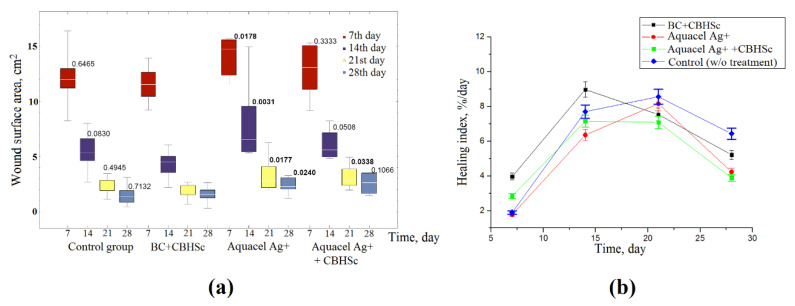
Box-plot of planimetric assessment data of wound surface area after different periods of observation (**a**) and healing indices (**b**) for experimental and control groups. The *p*-values are provided for comparison between the highlighted and BC + CBHSc groups in the given day of observation.

Neither positive nor negative differences between the control (without treatment) and BC + CBHSc group were observed (Figure 10). However, significant positive differences were seen when comparing the effect of BC + CBHSc and Aquacel Ag+ dressings on the wound surface area (Figure 10a). Analysis of the healing indices also revealed positive dynamics for the BC + CBHSc group at the 7th and maximum at the 14th day of the experiment, while for other groups it was observed closer to the 21st day. Comparison of data between groups with similar re-traumatization revealed an increase of healing process speed.

## 4. Discussion

The main goal of this study was to understand what changes occur in crystal and supramolecular structure of bacterial cellulose during the enzymatic treatment. In our opinion, such information can become the basis for both the development of new materials based on BC with an enzymatically changed structure or for wound dressings with controlled biodegradation. The actual requirements for wound dressing are quite extensive: it should provide a moist environment, thermal insulation and effective oxygen circulation, fluid drainage and epithelial migration, and assist in the absorption of wound exudate. The ideal dressing should protect wounds from primary and secondary bacterial infections. It should also be easily applied and removed painlessly, be biocompatible, and not cause allergic reactions [66,67]. An additional important requirement for biomaterials in tissue engineering is the ability to change their shape at a rate consistent with the formation of a new tissue [15]. Thus, control of the biodegradation speed is needed to avoid inhibition of wound healing and to damage a new epithelium.

Spontaneous cellulose hydrolysis can be achieved in several million years while well-documented enzymatic action on glycosidic linkages in polysaccharides accelerates degradation by up to 10^17^ times [68]. It mainly takes place under mild reaction conditions (neutral or near neutral pH and moderate temperature) [16,17,18,19]. However, traditionally the use of acids or alkalis is required for cellulose hydrolysis that is impossible under conditions of skin wound damage. On the other hand, enzyme action can be easily controlled using selected biocatalyst doses, temperature, or pH, depending on a goal. In this work, the conditions of CBHSc-catalyzed hydrolysis of BC pieces were selected based on the obtained kinetics and the requirements for models of wound healing considering clinical practice [69]. As expected, we did not observe the release of glucose equivalents in the untreated BC sample under selected conditions (data not shown) confirming the reported low biodegradability of BC in the absence of the enzyme.

We found that a change in the micromorphology of the surface of bacterial cellulose included the broadening of the nanoribbons comprising the 3D polymer network of BC, an increase in the distance between them, and formation and accumulation of submicron particles, apparently formed from the polysaccharide hydrolysis products, in the cells of the polymer network (Figure 7 and Figure 8, Table 3). These changes in the structural properties may have significant implications on the properties of BC-based material such as mechanical strength, elasticity, adhesion, permeability that are important for its use as a potential wound dressing.

One of the reasons why bacterial cellulose is actively used in the engineering of materials for skin and bone regeneration is its morphological similarity to collagen, which is the main protein of animal connective tissue with a three-dimensional nanoscale fibrillar structure [70]. However, the native collagen fibrils (95–175 nm in diameter and 300–500 nm in length) are larger than those of the native bacterial cellulose (8 nm thickness, 50 nm width) and have cylindrical shape [71,72]. The fibers of native BC have lamellar shape, as we confirm in this work by the combination of methods of SANS, USANS, and XRD. We showed that CBHSc-catalyzed hydrolysis affected the size of BC nanoribbons, increasing their thickness almost two-fold (up to 14 nm) and their width more than six-fold (up to 300 nm) after 24 h of treatment (according to SANS data, Table 2). These changes led to approaching the sizes of the components of BC polymer matrix to those of the natural collagen.

One of the most important criteria for choosing materials for wound dressing is permeability to steam and water, as well as variable adhesion to the skin, which is important to avoid repeated injury of the surface during wound healing [4,14,15,66,67]. We showed that enzymatic degradation of BC resulted in a substantial loosening of the polymer network, leading to a two-fold increase in the average pore diameter (from 2.2 to 4.7 nm) after 24 h of enzymatic treatment (Table 1). Such changes are expected to increase the permeability of the material to vapor and water, which may accelerate the regeneration process. At the same time, the specific surface of samples reduced by half relative to the native sample (from 260 to 100 m^2^·g^−1^, Table 1), which may lead to the change in the material adhesive properties, reducing its adhesion to the skin and thereby simplifying the use of BC-based dressings in regenerative medicine for the treatment of ulcers, burns, and wounds.

In this work, we used the fungal enzyme cellobiohydrolase for biodegradation of BC. We hypothesized that the inherent glycosylation of such proteins and hydrolytic product (cellobiose) release from BC into biological system may be factors in triggering an immune response [73]. For this reason, we tested the biocompatibility of the enzyme using primary human glioblastoma available in local cell collection [46]. Glioblastoma cell line was chosen due to its highest inherent sensitivity towards toxic effects of a wide compound panel checked previously (data not shown). Both cellobiohydrolase from *Scytalidium candidum* 3C used in our study and the hydrolysis products formed during biodegradation appear to be non-toxic (Figure 9). MTT-test showed no significant differences between the control, BC, CBHSc, and BC+CBHSc groups within four days. The data obtained in in vivo tests enable us to state that the standard treatment scheme (with weekly wound dressings replacement) did not lead to a sound atraumatic effect in all groups. However, the wound healing dynamics for the first two weeks and absolute surface area results were clearly better by the end of the second week and during further observation period differed significantly for the rats in BC+CBHSc group compared to the Aquacel Ag+ experimental group. No significant differences neither with the control group nor for the Aquacel Ag+ +CBHSc group were established. It should be noted that the control group did not receive any treatment, and as a result, was not traumatized during the wound healing.

In conclusion, the biodegradation catalyzed by cellobiohydrolase from *S. candidum* 3C affected both the crystalline and supramolecular structures (at the meso- and microscales) of bacterial cellulose. The addition of the enzyme to the BC samples did not lead to toxic effects in in vitro system and showed overall CBHSc-BC dressing safety in in vivo experiments. In our opinion, the results obtained can serve as a basis for further development of effective biodegradable dressings for the treatment of skin defects of various etiologies.

## Figures and Tables

**Table 1:** Texture parameters of supercritically dried native NGF and CBHSc–treated BC samples.

Time *t* of Biodegradation (min)	0	120	210	240	1440
*S*_BET_ (m^2^·g^−1^)	261 ± 29	191 ± 8	157 ± 6	189 ± 8	104 ± 4
*V*_P/P0__→0.99_ (cm^3^·g^−1^)	0.86	0.82	0.50	0.81	0.42
*d* (nm)	2.24	2.83	3.32	3.91	4.69

**Table 2:** Parameters of the mesostructure of the supercritically dried samples of BC after the biodegradation under the action of cellobiohydrolase from *S. candidum* 3C derived from the analysis of ultra-small angle neutron scattering (USANS) and small angle neutron scattering (SANS) data.

Time *t* of Biodegradation (min)	0	120	210	240	1440
*G*_2_^·^10^−2^, cm^−1^nm^−*s*2^	11.1 ± 1.3	7.3 ± 0.7	14.4 ± 1.9	-	-
*s*_2_	1.65 ± 0.02	1.64 ± 0.02	1.57 ± 0.02	-	-
*W*, nm	49 ± 5	56 ± 5	64 ± 6	-	-
*G*_1_^·^10^−3^, cm^−1^nm^−*s1*^	3.0 ± 0.1	4.3 ± 0.2	4.9 ± 0.4	9.3 ± 0.9	12.4 ± 1.5
*s*_1_	2.26 ± 0.02	2.10 ± 0.02	2.12 ± 0.02	2.02 ± 0.02	1.98 ± 0.05
*T*, nm	8.4 ± 0.3	10.1 ± 0.3	10.8 ± 0.5	12.6 ± 0.8	13.8 ± 1.5
*B*_1_^·^10^−5^, cm^−1^nm^−*n*^	1.0 ± 0.14	1.0 ± 0.16	2.0 ± 0.2	1.5 ± 0.2	3.0 ± 0.4
*D*_S_ = 6 − *n*	2.37 ± 0.03	2.51 ± 0.03	2.52 ± 0.02	2.49 ± 0.05	2.63 ± 0.04
*I*_inc_^·^10^−2^, cm^−1^	2.6 ± 0.1	1.6 ± 0.1	2.2 ± 0.1	2.9 ± 0.2	3.3 ± 0.2

**Table 3:** Roughness parameters obtained from AFM topologies.

	Critical Drying Time of BC, min			
	0	120	240	1440
*Ra*, nm				
18 μm × 18 μm	19.83		33.38	12.34
10 μm × 10 μm		14.49		
3.2 μm × 3.2 μm	11.15		13.37	5.81
2.5 μm × 2.5 μm		8.93 (11.13/6.08)		
*Rq*, nm				
18 μm × 18 μm	24.59		44.60	16.85
10 μm × 10 μm		19.00		
3.2 μm × 3.2 μm	13.74		17.13	7.66
2.5 μm × 2.5 μm		11.30 (13.66/7.60)		

*Ra* is the arithmetic average of the absolute values of the surface height deviations measured from the mean plane and *Rq* is the root mean square average of height deviation taken from the mean image data plane.
